# Low availability of code in ecology: A call for urgent action

**DOI:** 10.1371/journal.pbio.3000763

**Published:** 2020-07-28

**Authors:** Antica Culina, Ilona van den Berg, Simon Evans, Alfredo Sánchez-Tójar

**Affiliations:** 1 Department of Animal Ecology, Netherlands Institute of Ecology, NIOO-KNAW, Wageningen, the Netherlands; 2 Vrije Universiteit Amsterdam, Amsterdam, the Netherlands; 3 Centre for Ecology and Conservation, University of Exeter, Cornwall Campus, Penryn, United Kingdom; 4 Department of Zoology, University of Oxford, Oxford, United Kingdom; 5 Department of Evolutionary Biology, Bielefeld University, Bielefeld, Germany

## Abstract

Access to analytical code is essential for transparent and reproducible research. We review the state of code availability in ecology using a random sample of 346 nonmolecular articles published between 2015 and 2019 under mandatory or encouraged code-sharing policies. Our results call for urgent action to increase code availability: only 27% of eligible articles were accompanied by code. In contrast, data were available for 79% of eligible articles, highlighting that code availability is an important limiting factor for computational reproducibility in ecology. Although the percentage of ecological journals with mandatory or encouraged code-sharing policies has increased considerably, from 15% in 2015 to 75% in 2020, our results show that code-sharing policies are not adhered to by most authors. We hope these results will encourage journals, institutions, funding agencies, and researchers to address this alarming situation.

## Abstract 2

Publication of the analytical code underlying a scientific study is increasingly expected or even mandated by journals, allowing others to reproduce the results. However, a survey of more than 300 recently published ecology papers finds the majority have no code publicly available, handicapping efforts to improve scientific transparency.

## Introduction

There is a growing appreciation of the need for increased transparency in science. This is informed by the benefits of open and transparent research practices [1–3] and by the alarming lack of both reproducibility (same results obtained using the same data and analytical steps [4]) and replicability (qualitatively similar results obtained using same analytical steps on different datasets [4,5]) of scientific findings [6–8]. Efforts to increase reproducibility have, to date, mostly focused on making research data open and, more recently, FAIR (Findable, Accessible, Interoperable, and Reusable; [9]). Indeed, publishing the data underlying a scientific finding is required by an increasing number of scientific journals and funding agencies. This has led to a considerable increase in the volume, although not necessarily the quality, of available research data [10–12].

Another major component of research, without which reproducibility is difficult to achieve, is the computer code (hereafter code) underlying research findings. Scientists routinely write code for processing raw data, conducting statistical analyses, simulating models, creating figures, and even generating computationally reproducible articles [13,14]. For many ecologists, writing customized code has become an essential part of research, regardless of whether they conduct laboratory, field-based, or purely theoretical research [13,15,16]. Publicly sharing the code underlying scientific findings helps others to understand the analyses, evaluate the study’s conclusions, reuse the code for future analyses, and it increases overall research transparency and reproducibility (e.g., [17]). This might be particularly important in ecology because this discipline commonly requires the use of sophisticated statistical models, yet ecologists are often inadequately trained in quantitative and statistical methods [18].

Across fields, journals are increasingly adopting guidelines or creating policies that require or encourage authors to make the code underlying their findings publicly available [13, 17]. Yet, to what extent researchers follow these guidelines is uncertain, as is the general availability of code in ecology. We address this, specifically focusing on analytical code (i.e., computer code used for statistical analyses and/or simulations). We focus on analytical code because this code is essential for computational reproducibility of quantitative results, and because other types of codes (e.g., data processing) are rarely provided. We randomly sampled 400 articles published between June 2015 and May 2019 in 14 ecological journals that, as of June 2015, had either a mandatory code-sharing policy or explicitly encouraged authors to make their code available upon publication (see [13]). We identified and scrutinized 346 nonmolecular articles conducting statistical analyses and/or simulations to evaluate (i) the extent of code availability in ecology, and whether code availability has increased over time; (ii) the adherence to code publishing practices supporting code findability, accessibility, and reusability; and (iii) the limits to computational reproducibility. Additionally, we reassessed the current percentage of ecological journals with mandatory or encouraged code-sharing policies (Supporting information can be accessed at https://asanchez-tojar.github.io/code_in_ecology/supporting_information.html).

## Where are we now?

Amongst 96 ecological journals originally assessed for code-sharing policies by Mislan and colleagues [13], the number of journals with mandatory or encouraged code-sharing policies has increased from 14 in 2015 (15%; [13]) to 72 in 2020 (75%; S1 Table in https://asanchez-tojar.github.io/code_in_ecology/supporting_information.html). This is an encouraging increase that implies that the importance of code-sharing is now widely recognized. However, the existence of code-sharing policies does not necessarily translate into code availability (see below).

### Low code availability

Our main objective was to determine what proportion of articles conducting some type of statistical analysis and/or simulations were accompanied by the underlying analytical code. In our dataset, most statistical software used (>ca 90%) were either command line interfaces (e.g., R, Python, SAS, SAS Institute, Cary, NC) or graphical user interfaces (GUIs) that allow users to extract the code or syntax of the analyses (e.g., JMP, SAS Institute, Cary, NC; SPSS, IBM Corp., Armonk, NY). For software for which we were unsure whether code or syntax of the analyses could be extracted, we expanded our definition of code to also include screenshot-based protocols or alike that would allow other researchers to reproduce the quantitative results, and if not provided, we categorized those articles as not providing code.

**Fig 1:**
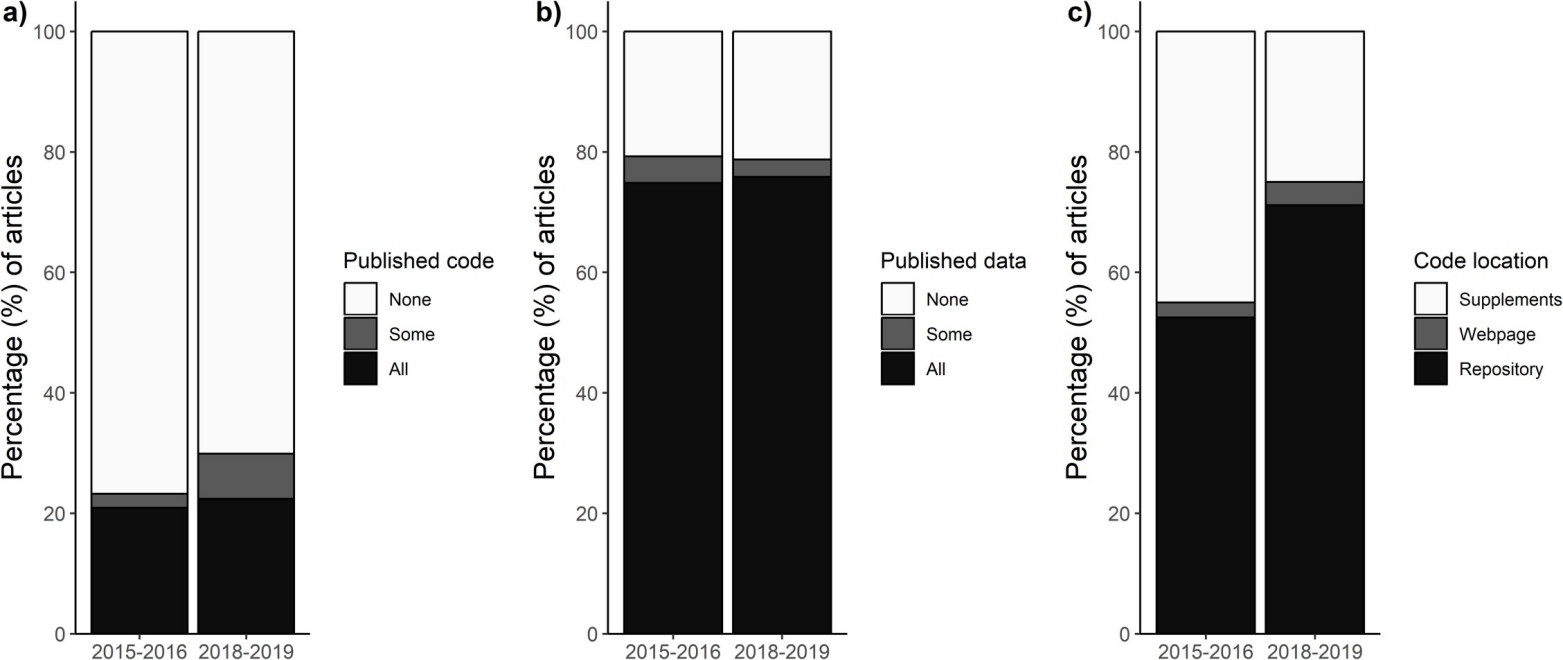
Code-sharing is at its infancy in ecology, whereas data-sharing seems more common. Percentage of articles reviewed that provided code (a) or data (b) for each of the time periods studied (2015–2016: 172 articles, 2018–2019: 174 articles). For studies that provided code (2015–2016: 40 articles, 2018–2019: 52 articles), the percentage of articles reviewed that hosted code in repositories (including nonpermanent platforms such GitHub; 2015–2016: 3 articles, 2018–2019: 8 articles), web pages, or supplements is shown (c). All percentages calculated in this manuscript and a description of how they were calculated are available in S2 Table in https://asanchez-tojar.github.io/code_in_ecology/supporting_information.html. Data to reproduce this figure are available at Culina and colleagues [19]: script “004_plotting.R”.

**Fig 2:**
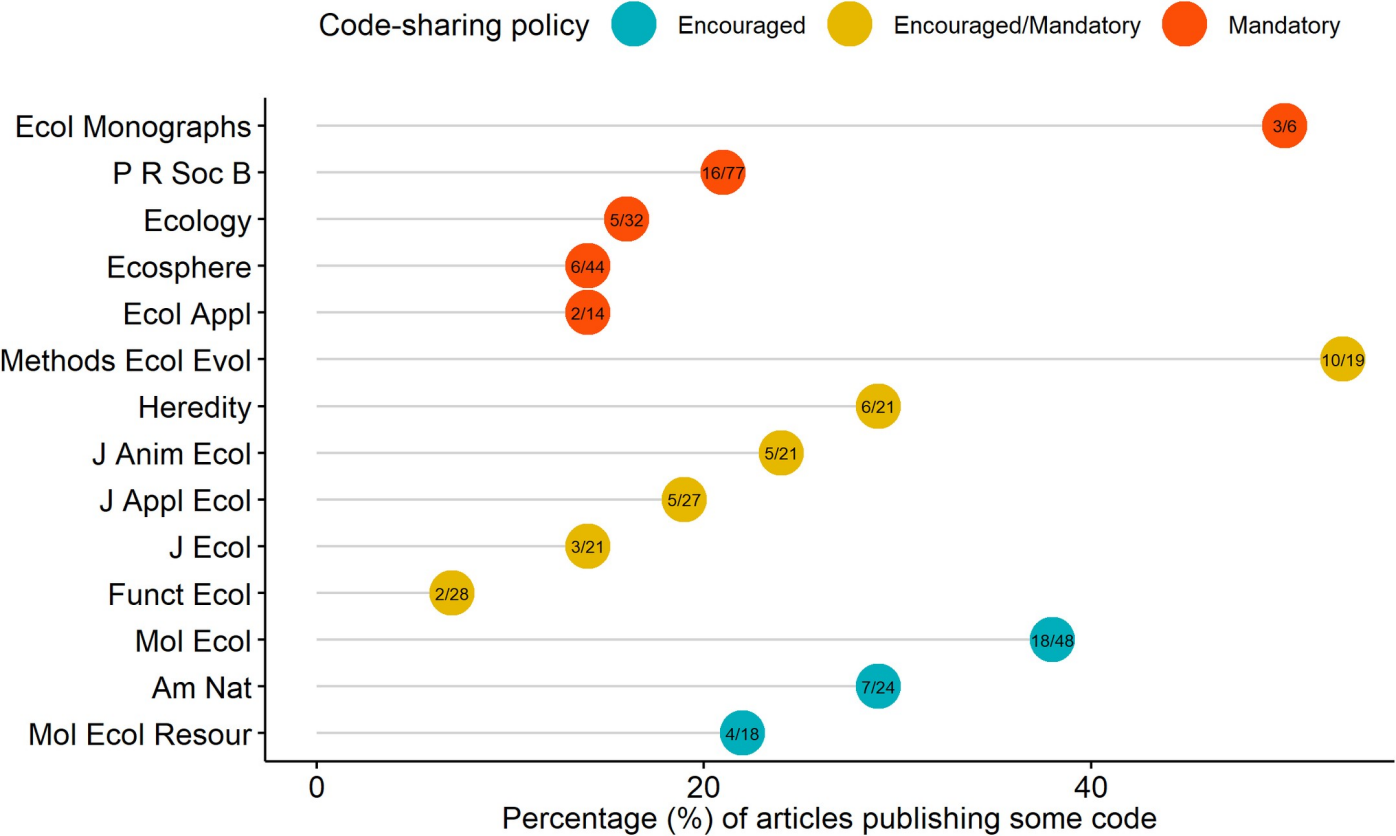
Estimated percentage of code-sharing for each of the 14 ecological journals reviewed. Journals are grouped by the strength of the code-sharing policy, and the ratio of articles with at least some published code to articles reviewed is shown within the circles. Full journal names are shown in S3 Table in https://asanchez-tojar.github.io/code_in_ecology/supporting_information.html. Data to reproduce this figure are available at Culina and colleagues [19]: script “004_plotting.R”.

We found that only 92 of 346 (27%) articles in our random sample of nonmolecular articles published between 2015 and 2019 were accompanied by either seemingly all (75 articles, 22%) or some (17 articles, 5%) of the code underlying the research findings. Code availability has slightly increased over the 5-year period studied here but remains alarmingly low (23% versus 30%, in 2015–2016 and 2018–2019, respectively; Fig 1A). Furthermore, the percentage of code available for each journal ranged between 7% and 53% (median = 22%, mean = 25%; Fig 2, S3 Table in https://asanchez-tojar.github.io/code_in_ecology/supporting_information.html), indicating that low code availability seems to be a general phenomenon and not driven by just a few journals. Given that the number of journals with a code-sharing policy has increased considerably over the last few years (see above), it seems likely that, overall, code availability in ecology has also increased. Nonetheless, the compliancy with the code-sharing policies lags behind (Figs 1A and 2).

### Findability, accessibility, and reusability

To be reused, code first needs to be found and accessed [20]. To be found, code availability should ideally be stated within the article or, if not, within the supplementary material of the article. In our dataset, 70 of the 92 articles (76%) that had code available enhanced “findability” by clearly referring to the availability of code within the text, mostly using the keywords “code” and/or “script” (96% of the articles that stated code availability). Code availability was most commonly stated in the “data accessibility” and/or “materials and methods” sections (94% of the articles that stated code availability), and only rarely in the “supplementary material” (4%) or the “discussion” section (1 article). Nonetheless, 24% of the articles that provided code did not explicitly state this code availability within the text. These articles commonly contained a general reference to the supplementary material and/or deposited data within the main text, but did not distinguish between code, primary data, and other supplementary materials, making code availability unclear to the reader.

We consider published code to be easily accessible when it is archived in ways that allow others to access it both now and in the foreseeable future. Our results indicate that there is some room for improvement in code accessibility because only 47 of the 92 articles (51%) that made their code available used online permanent repositories such as the Dryad Digital Repository, and 11 (12%) used exclusively GitHub, which is a nonpermanent, version controlled platform. A relatively large proportion of papers (34%) published their code as a supplementary material of the article. While archiving code as supplementary material does, in principle, support long-term access, not all code will be openly accessible without a journal subscription, which can reduce code accessibility. Furthermore, unlike dedicated repositories, there is no curation service in supplementary materials to ensure that files remain readable into the future and that the archive itself is maintained. Similarly, the use of nonpermanent, version controlled platforms such as GitHub should ideally be combined with publishing the code in a permanent repository (e.g., Zenodo) to protect long-term access. Overall, there has been some improvement in code publishing practices over time, with permanent repositories and GitHub gaining popularity in ecology (52% in 2015/2016 versus 71% in 2018/2019; Fig 1C). Given that many journals provide authors with free archiving of data and code in digital repositories, and given that free-of-charge archiving services such as Zenodo—which can be easily linked to GitHub—exist, we recommend that authors make their code available using these (and alike) services to enhance the long-term accessibility of their code. Furthermore, these services allow assignment of a digital object identifier (doi) to the code’s online location, which in combination with the correct license [21] makes the code more easily citable and reusable.

Finally, we explored code reusability. Code reusability depends on the complexity and documentation of the code, the software used to run the code, and the user’s own experience (e.g., [22]). We opted to simply evaluate whether the published code had some form of documentation (e.g., README), either as an accompanying document or embedded at the beginning of the code, and whether the code had inline comments explaining parts of the code. All code in our sample provided either one or both of these, but the level of elaboration ranged from detailed REAMDE files and/or comments to very minimal inline comments.

### Limits to computational reproducibility

Kitzes and colleagues [23] state that computational reproducibility is achieved when “a second investigator (including the original researcher in the future) can recreate the final reported results of the project, including key quantitative findings, tables, and figures, given only a set of files and written instructions.” Pragmatically, such a definition requires that the data (if any used) and code are available, a requirement met by a worryingly small percentage (21%) of the articles we reviewed (i.e., articles that provided both seemingly all data [if any used] and seemingly all code). Furthermore, the potential for computational reproducibility has not improved over time (20% in 2015/2016 versus 21% in 2018/2019).

**Fig 3:**
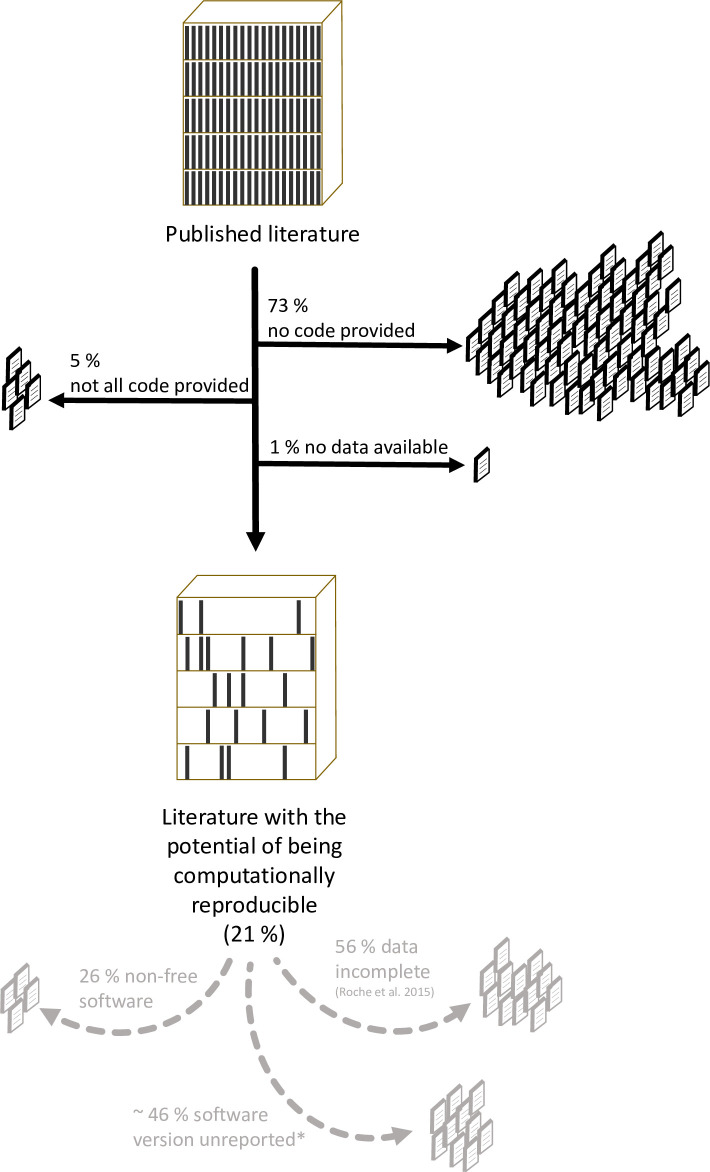
Depletion from published literature to literature with the potential of being computationally reproducible. Percentages estimated from a review of articles (*n* = 346) published in 14 ecological journals with mandatory or encouraged code-sharing policies, and, thus, these percentages likely overestimate the true percentage of computationally reproducible literature. Furthermore, because much code (and data) is published in nonpermanent repositories (see section “Findability, accessibility, and reusability”), long-term computational reproducibility is likely substantially lower. *Rough estimate based on the subset of articles published in 2018–2019. Data to reproduce this figure are available at [19].

Importantly, this figure (21%) is likely an overestimate. First, as highlighted above, we expect the real percentage of sharing to be lowered when also considering journals that do not require or encourage code (and data) sharing. Second, while data availability in our sample was comparatively high (263 out of 333 articles [79%] that used data shared at least some data, including seven embargoes; Fig 1B), this is likely an overestimation of the datasets that are complete and can be fully reused. Indeed, Roche and colleagues [11] recently found that 56% of archived datasets in ecology and evolution were incomplete. Furthermore, recent attempts at estimating true computational reproducibility of published articles within ecology [24] and other fields have shown that the percentage of true computational reproducibility ranged from 18% to 80% [25–28], even when data and code are provided (sometimes via author correspondence). Third, the use of non-free (i.e., proprietary) software can also be an impediment to computational reproducibility [26]. That said, most articles in our sample (74%) used exclusively nonproprietary software, with R being the most popular software (79% of these articles; note that 34 articles [10%] did not state the software used). Last, although we did not quantify this precisely, we noted that many articles failed to provide the version of the software and packages used, which can reduce computational reproducibility substantially [29]. While we did not aim to estimate true computational reproducibility of the research findings (i.e., by running the code on the data), it is alarming that probably fewer than 21% of published research articles in ecology are computationally reproducible, with multiple factors contributing to this low proportion (Fig 3).

## Where do we go from here?

The message of our review is clear: code availability in ecology is alarmingly low and, despite the existence of strong policies and guidelines in some journals, represents a major impediment to computational reproducibility of published research (Fig 3). On the one hand, there is no obvious obstacle preventing ecologists embracing the benefits of code-sharing, particularly given that ecology has experienced the shift from reliance on GUI-based statistical tools to a predominant reliance on custom-written scripts, particularly R ([16], this study). As [15] states, “If your code is good enough to do the job, then it is good enough to release—and releasing it will help your research and your field.” Our References section contains a list of several easy-to-follow guidelines on how to write and publish your code [15, 22, 30–34] and some more in-depth guidelines [35–38]. However, it seems clear that the collective benefit of code availability is insufficient encouragement to ensure good practice is widely adopted by authors, such that code sharing must be mandatory (or strongly encouraged). Journals, institutions, and funding agencies need to instigate and improve explicit code publishing policies (e.g., based on Transparency and Openness Promotion [TOP] Guidelines, https://www.cos.io/top) and to ensure these are followed by authors. For example, we noticed that it is often not easy to determine whether a journal mandates or merely encourages code sharing, which might contribute to a general failure on the part of authors to meet conditions of publication. Improving the current situation will require a concerted effort from funders, authors, reviewers, editors, and journals, not only to enforce sharing but also to increase incentives and provide relevant training to enable and encourage code- (and data-) sharing practices (see [15, 39–41]). We believe that access to code should be treated equivalently to access to primary data, i.e., as an essential (and obligatory) part of a publication in ecology.

## Abbreviations

doi: digital object identifier
FAIR: Findable, Accessible, Interoperable, and Reusable
GUI: graphical user interface
TOP Guidelines: Transparency and Openness Promotion Guidelines
