# Correction: Low availability of code in ecology: A call for urgent action

**DOI:** 10.1371/journal.pbio.3001048

**Published:** 2020-12-09

**Authors:** The PLOS Biology Staff

The Data Availability Statement is missing from this paper. The publisher apologizes for this error. The correct Data Availability Statement is as follows: Data and code to reproduce the results are available from https://doi.org/10.5281/zenodo.3833928. The Supporting Information can be accessed at https://asanchez-tojar.github.io/code_in_ecology/supporting_information.html.
